# Association Between Household Deprivation and Living in Informal Settlements and Incidence of Diarrhea in Children Under 5 in Eleven Latin American Cities

**DOI:** 10.1007/s11524-024-00854-y

**Published:** 2024-04-23

**Authors:** Victoria Alpaugh, Ana Ortigoza, Ariela Braverman Bronstein, Carolina Pérez-Ferrer, Nicolle Wagner-Gutierrez, Natalia Pacifico, Alex Ezeh, Waleska Teixeira Caiaffa, Gina Lovasi, Usama Bilal

**Affiliations:** 1 Urban Health Collaborative, Drexel Dornsife School of Public Health, 3600 Market St. Suite 730, Philadelphia, PA 19104 USA; 2 Department of Epidemiology and Biostatistics, Drexel Dornsife School of Public Health, 3600 Market St. Suite 730, Philadelphia, PA 19104 USA; 3 Department of Social and Environmental Determinants of Health Equity, Pan American Health Organization, Washington, D.C. USA; 4 Institute for Community Health, Cambridge Health Alliance, Malden, MA USA; 5 Center for Research in Population Health, National Institute of Public Health, Cuernavaca, Morelos Mexico; 6 Universidad de los Andes, School of Medicine, Bogotá, Colombia; 7 Institute of Collective Health, National University of Lanús, Remedios de Escalada, Argentina; 8 FJ Muñiz Infectious Hospital, Buenos Aires, Argentina; 9 Department of Community Health and Prevention, Drexel Dornsife School of Public Health, Philadelphia, PA USA; 10 Observatory for Urban Health in Belo Horizonte, School of Medicine, Federal University of Minas Gerais, Belo Horizonte, Brazil

**Keywords:** Childhood diarrhea, Informal settlements, Latin America, WaSH, Urban health, Global South

## Abstract

Diarrhea is a leading cause of death in children globally, mostly due to inadequate sanitary conditions and overcrowding. Poor housing quality and lack of tenure security that characterize informal settlements are key underlying contributors to these risk factors for childhood diarrhea deaths. The objective of this study is to better understand the physical attributes of informal settlement households in Latin American cities that are associated with childhood diarrhea. We used data from a household survey (Encuesta CAF) conducted by the Corporación Andina de Fomento (CAF), using responses from sampled individuals in eleven cities. We created a household deprivation score based on household water and sewage infrastructure, overcrowding, flooring and wall material, and security of tenure. We fitted a multivariable logistic regression model to estimate odds ratios (OR) and 95% confidence intervals (95% CI) to test the association between the deprivation score and its individual components and childhood diarrhea during the prior 2 weeks. We included a total of 4732 households with children, out of which 12.2% had diarrhea in the 2-week period prior to completing the survey. After adjusting for respondent age, gender, and city, we found a higher risk of diarrhea associated with higher household deprivation scores. Specifically, we found that the odds of diarrhea for children living in a mild and severe deprived household were 1.04 (95% CI 0.84–1.28) and 3.19 times (95% CI 1.80–5.63) higher, respectively, in comparison to households with no deprivation. These results highlight the connections between childhood health and deprived living conditions common in informal settlements.

**Supplementary Information:**

The online version contains supplementary material available at 10.1007/s11524-024-00854-y.

## Introduction

Diarrhea in children under 5 years old is a commonly used indicator of child, household, and community health and is also used to measure impact of public health interventions [1]. In 2017, diarrhea was the fifth leading cause of death in children under five, with this age group accounting for over a quarter of the 1.7 million diarrhea deaths globally [1]. The most studied risk factors for diarrhea include lack of water and sanitation, which were estimated to cause 502,000 and 280,000 of global diarrhea deaths respectively in 2012 [2]. Recent research suggests a variety of other socioeconomic and environmental factors that are associated with childhood diarrhea, such as household wealth, parent educational level and employment status, health facility coverage, waste disposal and collection, electricity, vaccination status, and household wall, roof, and floor material [2–7].

Many of these factors are related to the infrastructure and environmental conditions that characterize informal settlements, which house one-quarter of the world’s urban population [8]. As defined by UN-Habitat, “slum households” suffer from lack of one or more of the following: improved water source, improved sanitation facility, sufficient living area, housing durability, and security of tenure [8]. Beyond this definition, there is very little consistency or differentiation in the use of the terms slum, informal settlement, self-built neighborhoods, or unplanned squatter area [9]. Individual countries and cities commonly define and designate informal settlements separate and inconsistently from the UN definition, making comparisons of health and outcomes across these communities difficult. [10]

Generally, these communities are known for their lack of property ownership, streets and infrastructure and for makeshift, non-permanent housing [11]. Beyond lack of infrastructure, these communities are also characterized by political disenfranchisement, concentrated poverty and lack of access to education, employment, and healthcare [10]. Children are particularly vulnerable to these exposures, with high rates of infectious diseases, malnutrition, accidents, and violence [12]. Unsafe drinking water, inadequate sanitation, and waste disposal and overcrowding provide reservoirs and vectors for infectious diseases, and lead to increased rates of diarrhea and stunting. [12]

In Latin America overall, it is estimated that nearly 21% of the urban population is living in households in informal settlements [13]. Of the countries considered within this study, there is a wide range in this proportion: Argentina at 14.7%, Bolivia at 49.9%, Brazil at 15.2%, Colombia at 28.5%, Ecuador at 17.1%, Mexico at 15.1%, Panama at 21.3%, Peru at 33.1%, and Venezuela at 35.85% in 2018 [14]. The issues stemming from concentrated poverty are most evident in rural areas and in urban informal settlements, leading to poor health and inequalities [15]. Living conditions in these informal communities pose dire questions and challenges to human rights and health equity. Prior research has often studied diarrhea and sanitary and housing exposures in urban areas, but few studies outside of Africa or Asia have examined informal settlements more specifically, particularly in Latin America. Specifically, examining household and community effects of the lack of infrastructure within informal communities could provide more informative policies to address these issues. The aim of this study is to better understand the physical attributes of informal settlement households in Latin American cities that are associated with childhood diarrhea.

## Methods

### Data Source

We used an annual population-based survey (Encuesta CAF) conducted by the *Corporación Andina de Fomento* (CAF). This annual survey, starting in 2008, is completed throughout eleven Latin American cities and consists of demographic and socioeconomic questions, as well as additional rotating question modules, at the household level [16]. All CAF interviews are completed face-to-face by one respondent reporting information for all individuals in a household. Location of the household was georeferenced using the address provided by respondents and the latitude and longitude recorded by the survey team [16]. Sampling followed a semi-probabilistic, multi-stage, stratified design which is further described elsewhere [16]. This study specifically used the 2016 survey, collected between November 2016 and January 2017, which also collected data on households in informal settlements. Informal settlements were defined as over fifty contiguous homes with characteristics of lack of title deed, lack of formal access to public water, electricity, and sanitation, and have building deficiencies.

### Study Setting

An overview of the surveyed cities populations and sample sizes are outlined in Appendix 1. Four of these eleven cities (Bogota, Buenos Aires, Caracas, and Fortaleza) include an oversampling of informal settlements and characterized households as whether they belong to formal or informal areas (see definition of these areas below) [16]. We included households where participants answered “yes” to having a child under the age of 5 years old and had no missing data for the six exposure and outcome variables.

### Variables

#### Exposure

For exposures, we created six deprivation indicators to best align with the UN-Habitat definition of informal settlements. The six deprivation indicators include water access, sanitation, sufficient living area, structural quality of floors, structural quality of walls, and security of tenure. Specific operationalization of these indicators is outlined in Appendix Table 2. In summary, we defined deprivation in the domain of water as no water in the dwelling but with access to a community tap, shared well, or tank truck; in sewage as presence of a toilet/restroom to a pit, river, or canal; in overcrowding as greater than 3 people per room in a dwelling; in flooring as floors made of earth, sand, cardboard, or planks; in walls as walls made of waste material, sheets of cardboard, asbestos or metal, planks, stones with mud, and clay/cane with bark/adobe; and in security of tenure as being a de facto occupant without permission or tenants without signed contract. Finally, we also constructed a household deprivation score by conducting a simple count of the six indicator domains, ranging from 0 (no deprivation) to 6 (most severe deprivation). Based on exploratory analysis, we created score cutoffs for no deprivation (0), mild deprivation (1–2 indicators of deprivation), and severe deprivation (3 + indicators of deprivation).

#### Outcome

The outcome of interest was the presence of at least one episode of diarrhea in any child under 5 within the household in 2 weeks previous to the respondent answering the survey, created as a binary (yes/no) variable. This variable used the question “In the last two weeks have any of them (children up to 5 years of age) suffered from any of the following diseases…diarrhea?”.

#### Covariates

Covariates that were used in the final models included age (continuous) and gender (binary) of the (adult) respondent and city (categorical). Age or gender of the children was not available in the survey. We adjusted for city given the large differences in deprivation and prevalence of diarrhea across cities, to ensure that our results can be interpreted as within-city inferences. Based on exploratory analysis and available data, we categorized variables as follows: age (20–29, 30–39, 40–49, 50 +), and gender (men/women). Of note, the respondent was not necessarily the main caregiver of the children and was chosen as someone between 20 and 60 years old who could answer questions regarding the household.

### Statistical Analysis

First, we conducted a descriptive analysis examining the distribution of respondents, and household characteristics by reported diarrhea. We also computed percentages of households with reported diarrhea by deprivation indicator variable and city.

Second, we fitted multivariable logistic regression models to examine the odds of diarrhea associated with each deprivation indicator or the deprivation summary score and the outcome for all households with children under five and to calculate odds ratios (ORs) and 95% confidence intervals (95% CIs). Three sets of models with increasing levels of adjustment were completed for both the individual deprivation indicators (separately in different models) and a final model with the deprivation summary score. Model 1 was a crude model without adjustments, model 2 adjusted for age and gender of the respondent, and model 3 adjusted for age and gender of the respondent and city. We did not use the survey weights in the survey as our intent was not in generating estimates of prevalence of diarrhea, but rather on associations.

Third, we also leveraged the oversampling of informal settlements in four of the cities (Bogota, Buenos Aires, Caracas, and Fortaleza) to examine whether the survey designation of informal settlements in these cities was associated with diarrhea in the same magnitude and direction as the deprivation summary score and its composing deprivation indicators explored in the whole sample. We conducted this sub-analysis in two steps: (1) we repeated the analysis in the second step above restricted to these four cities (Bogotá, Buenos Aires, Caracas, and Fortaleza) to generate measures of association for the deprivation indicators that only include these cities; and (2) we then compared these results to an analysis where we used an indicator variable for living in the survey-defined informal settlement areas. These were defined as 50 or more contiguous houses that lack title deeds, lack formal access to public water, electricity, and sanitation, and have deficiencies of building [16]. The survey designation of an informal settlement area was only available for these four cities and was included in the survey as a household question designating formal, informal intraurban, and informal periphery. This variable was utilized as a binary formal and informal (including informal intraurban and informal periphery), included in a model without any of the other deprivation indicators or scores.

All data handling and statistical analysis was completed within SAS 9.4.

## Results

Overall, approximately 63% of the household respondents were female and 12% of households had at least one child under 5 with at least one episode of diarrhea within the last two weeks of the survey (Table 1). Rates of diarrhea were higher in households with younger respondents (20–29 and 30–39). Mexico City and Buenos Aires had the lowest percentage of households with diarrhea cases (2.0% and 6.7%, respectively). La Paz and Caracas had the highest percentage of households with diarrhea cases (22.9% and 20.3% respectively). A smaller proportion of households had deprivation in the domains of water (1.31%), overcrowding (4.37%), flooring (4.35%), and walls (5.39%), while a larger proportion households had deprivation in the domains of sewage (7.42%) and security of tenure (10.06%) (Appendix Table 3).

**Table 1:** Study population demographics

	Total	No diarrhea	Diarrhea
Sample size*	**4732**	**87.8% (4154)**	**12.2% (578)**
Gender (male)**	37.3% (1766)	87.8% (1550)	12.2% (216)
Age, years**			
20–29	37.5% (1772)	86.0% (1523)	14.0% (249)
30–39	34.4% (1628)	87.0% (1416)	13.0% (212)
40–49	21.4% (1013)	92.1% (933)	7.9% (80)
50 +	6.7% (319)	88.4% (282)	11.6% (37)
City			
Bogota	8.9% (420)	85.2% (358)	14.8% (62)
Lima	9.0% (427)	88.8% (379)	11.2% (48)
Mexico City	15.0% (709)	98.0% (695)	2.0% (14)
Fortaleza	9.9% (466)	83.0% (387)	17.0% (79)
Buenos Aires	12.3% (580)	93.3% (541)	6.7% (39)
Panama	8.3% (394)	87.8% (346)	12.2% (48)
San Paulo	5.9% (281)	91.1% (256)	8.9% (25)
La Paz	8.0% (380)	77.1% (293)	22.9% (87)
Quito	7.4% (349)	86.5% (302)	13.5% (47)
Montevideo	5.6% (267)	86.5% (231)	13.5% (36)
Caracas	9.7% (459)	79.7% (366)	20.3% (93)
Deprivation indicator			
Water	1.31% (62)	83.87% (52)	16.13% (10)
Sewage	7.42% (351)	84.62% (297)	15.38% (54)
Overcrowding	4.37% (207)	84.54% (175)	15.46% (32)
Flooring	4.35% (206)	82.52% (170)	17.48% (36)
Walls	5.39% (255)	79.61% (203)	20.39% (52)
Security of tenure	10.06% (476)	85.29% (406)	14.71% (70)
Deprivation summary score			
No deprivation (0 exposures)	75.3% (3563)	88.72% (3161)	11.28% (402)
Mild deprivation (1–2 exposures)	23.42% (1108)	85.83% (951)	14.17% (157)
Severe deprivation (3 + exposures)	1.29% (61)	68.85% (42)	31.15% (19)

^*^Survey respondent, one per household. **All answered in terms of respondent (interviewee gender and age)

All single deprivation indicators were associated with greater odds of diarrhea in our fully adjusted model (Table 2, model 3), ranging from 2 to 55% higher odds; however, the only indicator of deprivation significantly associated with diarrhea was the lack of durable walls (OR = 1.55, 95% CI 1.11–2.17). Walls and water indicators had the strongest association with diarrhea by effect size, while flooring, sanitation, and security of tenure had the weakest association. We also found a linear gradient between the deprivation summary score and the odds of diarrhea. In the fully adjusted model, respondents living in a household with mild deprivation had 1.04 times the odds of diarrhea (95% CI 0.84–1.28) and those living in households with severe deprivation had 3.19 (95% CI 1.80–5.63) higher odds of a child under 5 having diarrhea within the last 2 weeks than those with no deprivation. Associations were stronger but consistent in the unadjusted models and in the models adjusted by age and gender only.

**Table 2:** Odds ratio of diarrhea associated with household deprivation score and each of its composing indicators in eleven Latin American cities (Bogota, Lima, Mexico City, Fortaleza, Buenos Aires, Panama, San Paulo, La Paz, Quito, Montevideo and Caracas)

Deprivation indicator**	Model 1 (unadjusted model)	Model 2 (adjusted for age and gender)	Model 3 (adjusted for age, gender and city)
Water deprivation*	1.39 (0.70–2.75)	1.35 (0.68–2.67)	1.48 (0.73–2.98)
Sanitation deprivation*	1.34 (0.99–1.81)	**1.36 (1.01–1.85)**	1.26 (0.90–1.76)
Crowding*	1.33 (0.91–1.96)	1.33 (0.90–1.96)	1.27 (0.85–1.88)
Flooring deprivation*	**1.56 (1.08–2.26)**	**1.64 (1.13–2.38)**	1.26 (0.86–1.85)
Walls deprivation*	**1.92 (1.40–2.64)**	**1.87 (1.36–2.57)**	**1.55 (1.11–2.17)**
Lack of tenure*	1.27 (0.97–1.67)	1.21 (0.92–1.59)	1.02 (0.77–1.35)
Deprivation score			
No deprivation (0 indicators of deprivation)	1 (ref.)	1 (ref.)	1 (ref.)
Mild deprivation (1–2 indicators of deprivation)	**1.30 (1.07–1.58)**	**1.27 (1.04–1.55)**	1.04 (0.84–1.28)
Severe deprivation (3 + indicators of deprivation)	**3.56 (2.05–6.18)**	**3.55 (2.04–6.17)**	**3.19 (1.80–5.63)**

^*^Reference value no deprivation for each exposure

^**^Water deprivation: no water in the house but access to community tap, shared well, or tank truck

Sewage deprivation: toilet/restroom to pit, river, or canal

Overcrowding deprivation: greater than 3 people per room in the household

Flooring deprivation: earth, sand, cardboard, or planks

Walls deprivation: waste material, sheet of cardboard, asbestos or metal, planks, stone with mud, clay/cane with bark/adobe

Security of tenure deprivation: de facto occupant without permission or tenants without a signed contract

Table 3 shows associations of diarrhea with the (a) deprivation indicators, (b) deprivation summary score, and (c) survey-designated informal settlements (see definition in the Methods section). Within the subset of four cities, associations with the deprivation indicators and the deprivation summary score with diarrhea were similar compared to the main analysis. However, we found no association of survey designation of informal settlement with childhood diarrhea (OR 1.01, 95% CI 0.77–1.32) after adjusting for age, gender, and city.

**Table 3:** Odds ratio of diarrhea associated with individual and summarized deprivation indicators and survey designation of informal settlement in 4 Latin American Cities (Bogota, Buenos Aires, Caracas and Fortaleza). All indicators were introduced in different models

Deprivation indicator**	Model 1 (unadjusted model)	Model 2 (adjusted for age and gender)	Model 3 (adjusted for age, gender, and city)
Survey designated informal settlement	1.01 (0.78–1.31)	0.96 (0.74–1.25)	1.01 (0.77–1.32)
Water*	1.74 (0.57–5.33)	1.85 (0.60–5.74)	2.55 (0.80–8.16)
Sanitation*	1.28 (0.89–1.84)	1.23 (0.85–1.78)	1.29 (0.86–1.95)
Crowding*	1.41 (0.78–2.56)	1.43 (0.79–2.62)	1.75 (0.94–3.25)
Flooring*	**1.88 (1.14–3.12)**	**1.84 (1.10–3.06)**	1.68 (0.99–2.84)
Walls*	**1.91 (1.11–3.28)**	**1.81 (1.04–3.13)**	**2.04 (1.15–3.63)**
Tenure*	1.11 (0.77–1.59)	1.02 (0.71–1.47)	0.97 (0.66–1.41)
No deprivation (0 exposures)	1 (ref.)	1 (ref.)	1 (ref.)
Mild deprivation (1–2 exposures)	1.18 (0.90–1.57)	1.11 (0.84–1.48)	1.15 (0.85–1.55)
Severe deprivation (3 + exposures)	**3.43 (1.68–7.02)**	**3.44 (1.67–7.11)**	**3.61 (1.71–7.63)**

^*^Reference value no deprivation for each indicator

^**^Water deprivation: no water in house but access to community tap, shared well or tank truck

Sewage deprivation: toilet/restroom to pit, river or canal

Overcrowding deprivation: greater than 3 people per room in household

Flooring deprivation: earth, sand, cardboard or planks

Walls deprivation: waste material, sheet of cardboard, asbestos or metal, planks, stone with mud, clay/cane with bark/adobe

Security of tenure deprivation: de facto occupant without permission or tenants without signed contract

## Discussion

The aim of this study was to better understand the physical attributes of households in Latin American cities that are associated with childhood diarrhea. We highlight three key findings. First, each indicator used to characterize household deprivation as proxy of informal settlements was associated with higher odds of diarrhea among children under five, although variability around these estimates was high. Second, we found that having multiple concurrent deprivation indicators was strongly associated with childhood diarrhea. Last, while our main results held for the four cities with oversampling of informal settlements, we did not find a significant association between living in a survey-designated informal settlement and childhood diarrhea.

While previous studies have examined the association between similar household living conditions and diarrhea in children five and younger, almost all of these are within Africa and Southeast Asia [1–7, 17–22] and several include or exclusively focus on informal settlements [1, 3, 4]. While there is some variety in the age range and outcome definitions, most of these studies include children under five who have had diarrhea within the last 2 weeks. Of those with that specific outcome definition aligning with this study, total diarrhea rates ranged from 11 to 27.8% [1–7, 18–23]. Although this study focuses on a different region than most available research, our findings had a comparable diarrhea rate of 12.2%.

While many available studies focus on the association between diarrhea and similar deprivation indicators to this study, many define and categorize these indicators differently. For water access, several studies looking at unimproved water source found a significant association with increased risk of childhood diarrhea [5, 6, 20, 21]. While this study did not find a significant association with the deprivation indicator of water alone, the magnitude of our associations is in line with those previously reported in the literature. Of note, a very small proportion of the participants had a categorization of deprivation for the water indicator. For sanitation, several studies looking at unimproved sanitation found varying results, with two studies finding no significant association and two with a small association with childhood diarrhea [2, 5, 21]. Our findings are in line with these previous studies. For overcrowding, two studies found significant associations with diarrhea with varying effect sizes, [19, 23] similar to ours. We identified four studies exploring the associations of flooring with diarrhea, but only one observed a significant association [5], similar to ours [5–7, 22]. The only study we found exploring wall durability reported a significant effect on the most similar model to ours (although it adjusted for stunting, which may move estimates toward the null) [5]. Interestingly, wall durability was the only variable for which we found a significant effect when examined in isolation, and our effect was stronger than the cited study. We found no other studies studying the security of tenure and childhood diarrhea.

We found a strong association in the models using the deprivation summary score combining the effects of multiple deprivations. Within the severe deprivation category, children were found to have three times the risk of diarrhea within the last 2 weeks compared to children in households with no deprivations. This suggests many significant health implications of having multiple household deprivations, which commonly align with those living within an informal settlement. The lack of basic infrastructure, including water, sanitation, and waste removal, in overcrowded urban areas can create conditions that increase the transmission of infectious diseases, particularly diarrheal diseases. [12] Additionally, inadequate investment in education, transportation, economic opportunity, and healthcare further exacerbates poor health outcomes in these areas [12].

Last, we also found that in the four cities for which an oversampling and designation of informal settlements were available, our deprivation summary score was still strongly associated with diarrhea, but the survey definition of informal settlements was not associated. Nguyen et al. (2021) found a difference of 13.4 to 21.2% in diarrhea between formal and informal settlements, respectively, while our case study did not find a significant difference (11.9 to 12.3%) [1]. Our findings indicate that household deprivations themselves, specifically multiple deprivations within one household, are more associated with of diarrhea than the general risk of living within an informal settlement. Some participants may live in households with many of the deprivation indicators but do not live in a community of at least 50 houses with the same risks, and therefore are not categorized as an informal settlement. Similarly, there may be people living in informal settlements without many of the deprivations we found to be associated with diarrhea. This issue is further complicated by the lack of consistency in defining informal settlements; other case studies finding a large gap between survey designated informal settlements and general areas of deprivation [24, 25]. Deprivations are found in many households outside of designated informal settlements and differences in living standards between regions of the world greatly impact the boundaries drawn defining these settlements [24]. This is an important policy implication; if only officially designated informal settlements are targeted by social programs, many other at-risk households with these deprivations may be left out.

This study provides an analysis on the associations between household deprivations and informal settlements on childhood diarrhea in Latin America. Because this region is highly urbanized and has many informal settlements, it is critical to understand the health implications of lacking infrastructure and concentrated poverty within these cities. Further and more extensive research is needed to investigate this complicated issue and the other outcomes that can come from these deprivations. One of the key limitations of the study is the outcome variable of diarrhea within the last 2 weeks, which is nonspecific to severity or cause, and could also be underreported by the survey respondent. However, this method aligns with the variable definition used in similar research on this topic [1–7, 17–22]. An additional limitation is that our deprivation indicators do not perfectly align with the UN-HABITAT definitions. While not perfectly aligned, our operationalization attempted to best fit these definitions, and we found the deprivation summary score to be highly predictive of diarrhea. Furthermore, we lacked data on the age of the child, and used instead age of the respondent as a proxy. We also lacked data on the relationship of the survey respondent to the children. The variable of number of children was available within the survey but was not used due to lack of information or specificity on children age, whether they live within the household or other children living in the household that are not those of the respondent.

## Conclusions

Lack of water and sanitation access, durability of household construction materials, overcrowding, and security of tenure are all defining parts of informal settlements. This study investigated how they individually, and in combination, are related to rates of childhood diarrhea. The results found that multiple deprivations together were highly predictive of childhood diarrhea, with children living in areas with severe deprivation having 3 times higher odds of diarrhea. These findings point to the important health implications of households having these deprivations in urban Latin American environments. These findings also point to the notion that improving child health requires considering housing, water, sanitation, and other social determinants of health, instead of just focusing on narrow preventive health programs. It is crucial for local and national governments to prioritize and commit to improving the living conditions in these communities to meet the human rights of those living in slums and improve child health outcomes. This may involve implementing policies and initiatives aimed at improving access to clean water and sanitation, slum upgrading, investing in healthcare and education infrastructure, and promoting economic development in these areas. [24, 26].

### Supplementary Information

Below is the link to the electronic supplementary material.

Supplementary file1 (DOCX 20 KB)

## Data Availability

For information on data availability please visit https://drexel.edu/lac/ and https://data.lacurbanhealth.org/.

## References

[1] Nguyen TYC, Fagbayigbo BO, Cissé G, et al. Diarrhoea among children aged under five years and risk factors in informal settlements: a cross-sectional study in Cape Town, South Africa. *Int J Environ Res Public Health.* 2021;18(11). 10.3390/ijerph18116043 PMC8199993 34199733

[2] Komarulzaman A, Smits J, de Jong E (2017). Clean water, sanitation and diarrhoea in Indonesia: effects of household and community factors. Glob Public Health.

[3] Dos Santos S, OuedraogoFde C, Soura AB (2015). Water-related factors and childhood diarrhoea in African informal settlements. A cross-sectional study in Ouagadougou (Burkina Faso). J Water Health.

[4] Adane M, Mengistie B, Kloos H, Medhin G, Mulat W (2017). Sanitation facilities, hygienic conditions, and prevalence of acute diarrhea among under-five children in slums of Addis Ababa, Ethiopia: baseline survey of a longitudinal study. PLoS One.

[5] Yaya S, Hudani A, Udenigwe O, Shah V, Ekholuenetale M, Bishwajit G. Improving water, sanitation and hygiene practices, and housing quality to prevent diarrhea among under-five children in Nigeria. *Trop Med Infect Dis.* 2018;3(2). 10.3390/tropicalmed3020041 PMC6073794 30274437

[6] SinmegnMihrete T, AsresAlemie G, Shimeka TA (2014). Determinants of childhood diarrhea among underfive children in Benishangul Gumuz Regional State. North West Ethiopia BMC Pediatr.

[7] Workie GY, Akalu TY, Baraki AG (2019). Environmental factors affecting childhood diarrheal disease among under-five children in Jamma district, South Wello zone, Northeast Ethiopia. BMC Infect Dis.

[8] SDG Indicator Metadata*.* United Nations; 2021. https://unstats.un.org/sdgs/metadata/files/Metadata-11-01-01.pdf.

[9] Peralta R. Lesson of hope: a case study on self-built homes in the informal neighborhoods of Tijuana. In: Marinic G, Meninato P, editors. *Informality and the city: theories, actions and interventions*. Cham: Springer International Publishing; 2022. pp. 275–87.

[10] Snyder RE, Jaimes G, Riley LW, Faerstein E, Corburn J (2014). A comparison of social and spatial determinants of health between formal and informal settlements in a large metropolitan setting in Brazil. J Urban Health.

[11] Corburn J, Sverdlik A. Informal settlements and human health. In: Nieuwenhuijsen M, Khreis H, editors. *Integrating human health into urban and transport planning*. Cham, Switzerland: Springer International Publishing; 2019.

[12] Ezeh A, Oyebode O, Satterthwaite D (2017). The history, geography, and sociology of slums and the health problems of people who live in slums. Lancet.

[13] Population living in slums (% of urban population) - Latin America & Caribbean. https://data.worldbank.org/indicator/EN.POP.SLUM.UR.ZS?locations=ZJ Accessed May 25, 2022.

[14] Site UD. Proportion of urban population living in slum households by country or area 1990–2018. https://data.unhabitat.org/datasets/proportion-of-urban-population-living-in-slum-households-by-country-or-area-1990-2018-percent/explore. Accessed 1 Mar 2022.

[15] Abramo L, Cecchini S, Ullmann H (2020). Addressing health inequalities in Latin America: the role of social protection. Cien Saude Colet.

[16] CAF Banco de Desarrollo de América Latina Encuesta. *Información metodológica ECAF*. 2016. Available online: https://www.caf.com/media/29899/informe-metodologico-ecaf-2016.pdf.

[17] Luby SP, Rahman M, Arnold BF (2018). Effects of water quality, sanitation, handwashing, and nutritional interventions on diarrhoea and child growth in rural Bangladesh: a cluster randomised controlled trial. Lancet Glob Health.

[18] Thiam S, Diene AN, Fuhrimann S (2017). Prevalence of diarrhoea and risk factors among children under five years old in Mbour, Senegal: a cross-sectional study. Infect Dis Poverty.

[19] Siziya S, Muula AS, Rudatsikira E (2013). Correlates of diarrhoea among children below the age of 5 years in Sudan. Afr Health Sci.

[20] Godana W, Mengistie B (2013). Determinants of acute diarrhoea among children under five years of age in Derashe District, Southern Ethiopia. Rural Remote Health.

[21] Natnael T, Lingerew M, Adane M (2021). Prevalence of acute diarrhea and associated factors among children under five in semi-urban areas of northeastern Ethiopia. BMC Pediatr.

[22] Sahiledengle B, Agho K (2021). Determinants of childhood diarrhea in households with improved water, sanitation, and hygiene (WASH) in Ethiopia: evidence from a repeated cross-sectional study. Environ Health Insights.

[23] Bahartha AS, AlEzzi JI (2015). Risk factors of diarrhea in children under 5 years in Al-Mukalla. Yemen Saudi Med J.

[24] Cities Alliance. *The challenge of slums – an overview of past approaches to tackle it*. Brussels, Belgium: Cities Alliance; 2021. https://www.citiesalliance.org/resources/publications/publications/challenge-slums—-overview-past-approaches-tackle-it.

[25] Lilford R, Kyobutungi C, Ndugwa R (2019). Because space matters: conceptual framework to help distinguish slum from non-slum urban areas. BMJ Glob Health.

[26] Lilford RJ, Oyebode O, Satterthwaite D (2017). Improving the health and welfare of people who live in slums. Lancet.

