# Supplementary material for: Association Between Household Deprivation and Living in Informal Settlements and Incidence of Diarrhea in Children Under 5 in Eleven Latin American Cities
Source: J Urban Health. 2024 Apr 23;101(3):629–37. doi: 10.1007/s11524-024-00854-y (PMC11189882; doi:10.1007/s11524-024-00854-y)
Supplement: Supplementary file 1 — Supplementary file1 (DOCX 20 KB) [file 11524_2024_854_MOESM1_ESM.docx]

**Appendix**

**Appendix Table 1: City population and sample size for each of the designated seven cities used in the study.**

| **City** | **Country** | **Population** | **Sample Size** |
| --- | --- | --- | --- |
| Bogota | Colombia | 7,181,469 | Formal: 1000 |
|  |  |  | Informal: 538 |
| Buenos Aires | Argentina | 15,567,820 | Formal: 1006 |
|  |  |  | Informal: 554 |
| Caracas | Venezuela | 2,082,130 | Formal: 1000 |
|  |  |  | Informal: 500 |
| Fortaleza | Brazil | City proper: 862,750 | Formal: 1100 |
|  |  | Urban agglomeration: 2,452,158 | Informal: 600 |
| La Paz | Bolivia | 789,585 | 1000 |
| Lima | Peru | 9,562,280 | 1001 |
| Mexico City | Mexico | 22,167,521 | 1006 |
| Montevideo | Uruguay | 1,380,432 | 1000 |
| Panama City | Panama | City proper: 501,477  Urban agglomeration: 1,153,170 | 600 |
| Quito | Ecuador | 1,619,146 | 1000 |
| Sao Paulo | Brazil | City proper: 105,269  Urban agglomeration: 11,152,968 | 1000 |

* Designation of sample size by formality is outlined for the three cities with a survey focus on informality.

<https://data.un.org/Data>

**Appendix Table 2: Indicators to define informal settlements**

| **Indicator** | **Question** | **Responses** | **Deprivation** | **No Deprivation** | **Aligned UN-Habitat Definition** |
| --- | --- | --- | --- | --- | --- |
| **Water Access** | Does this house access water mainly because… | (1) formal connection to public network/aqueduct, (2) water well in the house (3) informal connection to the public network (4) connection to the public network through a neighbor (5) no water in the house but access to tap/community spout (6) shared water well (7) tank truck | **5-7** | **1-4** | **Access to improved water:** Piped water into dwelling, plot or yard; public tap/stand pipe serving no more than 5 households; protected spring; rainwater collection; bottled water (if secondary source is also improved); bore hole/tube well; and, protected dug well |
| **Sanitation** | What drainage service (excreta disposal) do you have? | 1) toilet/bathroom to public sewage system/sewer (2) toilet/bathroom to well with septic tank (3) toilet/restroom to pit, excavation in the ground (4) toilet/restroom to river, canal | **3-4** | **1-2** | **Access to improved sanitation:** flush/pour-flush toilets or latrines connected to a sewer, septic tank or pit; ventilated improved pit latrine; pit latrine with a slab or platform, which covers the pit entirely; and, composting toilets/latrines. |
| **Sufficient Living Area** | “How many rooms does this home have for its exclusive use, excluding bathrooms?” and “How many people make up your home? | Computed number of people / number of rooms | **>3** | **<=3** | **Sufficient living area:** not more than three people share the same habitable room |
| **Structural Quality - floors** | “What is the predominant floor construction material?” | (1) earth, sand, cardboard or planks (2) cement or gravel (3) mosaic, granite, marble, ceramic, terracotta, parquet, carpet or time | **1** | **2-3** | **Structural quality:** permanency of structure (permanent building material for the walls, roof and floor; compliance with building codes; the dwelling is not in a dilapidated state; the dwelling is not in need of major repair); and location of house (hazardous location; the dwelling is not located on or near toxic waste; the dwelling is not located in a flood plain; the dwelling is not located on a steep slope; the dwelling is not located in a dangerous right of way: rail, highway, airport, power lines |
| **Structural Quality - walls** | “What is the predominant material of the exterior walls?” | (1) waste material or cardboard sheet (2) sheet of asbestos or metal/sheets/zinc (3) planks, stone with mud, clay/cane with bark/adobe (4b) brick, stone, cement block or concrete | **1-3** | **4** |  |
| **Security of Tenure** | “You or the members of this household are:” & | (1) owners of the dwelling and land (2) owners of the dwelling only (3) free occupants with permission (4) de facto occupants (without permission) (5) tenants of whole house (6) tenants of the housing part | **4 (and 5-6 if no signed contract)** | **1-3 (and 5-6 if have signed contract)** | **Security of Tenure:** there is evidence of documentation that can be used as proof of secure tenure status; and, there is either de facto or perceived protection from forced evictions |
|  | “Do you have a signed contract for the rental of this home?” | (1) yes (2) no |  |  |  |

(SDG indicator metadata - United Nations

**Appendix Table 3: Prevalence of each deprivation indicator and summary category**

|  | **Total** | |
| --- | --- | --- |
|  | No deprivation | Deprivation |
| Water | 98.69% (4670) | 1.31% (62) |
| Sewage | 92.58% (4381) | 7.42% (351) |
| Overcrowding | 95.63% (4525) | 4.37% (207) |
| Flooring | 95.65% (4526) | 4.35% (206) |
| Walls | 94.61% (4477) | 5.39% (255) |
| Security of Tenure | 89.94% (4256) | 10.06% (476) |
| **No Deprivation**  (0 exposures) | 75.30% (3563) | |
| **Mild deprivation**  (1-2 exposures) | 23.42% (1108) | |
| **Severe deprivation**  (3+ exposures) | 1.29% (61) | |
